# Surface immunogenic protein from Streptococcus agalactiae and *Fissurella latimarginata* hemocyanin are TLR4 ligands and activate MyD88- and TRIF dependent signaling pathways

**DOI:** 10.3389/fimmu.2023.1186188

**Published:** 2023-09-18

**Authors:** Diego A. Díaz-Dinamarca, Michelle L. Salazar, Daniel F. Escobar, Byron N. Castillo, Bastián Valdebenito, Pablo Díaz, Augusto Manubens, Fabián Salazar, Mayarling F. Troncoso, Sergio Lavandero, Janepsy Díaz, María Inés Becker, Abel E. Vásquez

**Affiliations:** ^1^ Sección de Biotecnología, Subdepartamento, Innovación, Desarrollo, Transferencia Tecnológica (I+D+T) y Evaluación de Tecnologías Sanitarias (ETESA), Instituto de Salud Pública, Santiago, Chile; ^2^ Laboratorio de Inmunología, Fundación Ciencia y Tecnología para el Desarrollo (FUCITED), Santiago, Chile; ^3^ Facultad de Ciencias Químicas y Farmacéuticas, Universidad de Chile, Santiago, Chile; ^4^ Investigación y Desarrollo, BIOSONDA S.A., Santiago, Chile; ^5^ Medical Research Council Centre for Medical Mycology, University of Exeter, Exeter, United Kingdom; ^6^ Advanced Center for Chronic Diseases (ACCDiS), Facultad Ciencias Químicas y Farmacéuticas and Facultad de Medicina, Universidad de Chile, Santiago, Chile; ^7^ Department of Internal Medicine (Cardiology Division), University of Texas Southwestern Medical Center, Dallas, TX, United States; ^8^ Departamento Agencia Nacional de Dispositivos Médicos, Innovación y Desarrollo, Instituto de Salud Pública de Chile, Santiago, Chile; ^9^ Facultad de Ciencias de la Salud, Escuela de Medicina, Universidad del Alba, Santiago, Chile

**Keywords:** protein-based adjuvants (PBAs), TLR4 agonist, MyD88, TRIF, antigen-presenting cells, vaccines, recombinant surface immunological protein from *Streptococcus agalactiae* (rSIP), hemocyanin from *Fissurella latimarginata* (FLH)

## Abstract

The development of vaccine adjuvants is of interest for the management of chronic diseases, cancer, and future pandemics. Therefore, the role of Toll-like receptors (TLRs) in the effects of vaccine adjuvants has been investigated. TLR4 ligand-based adjuvants are the most frequently used adjuvants for human vaccines. Among TLR family members, TLR4 has unique dual signaling capabilities due to the recruitment of two adapter proteins, myeloid differentiation marker 88 (MyD88) and interferon-β adapter inducer containing the toll-interleukin-1 receptor (TIR) domain (TRIF). MyD88-mediated signaling triggers a proinflammatory innate immune response, while TRIF-mediated signaling leads to an adaptive immune response. Most studies have used lipopolysaccharide-based ligands as TLR4 ligand-based adjuvants; however, although protein-based ligands have been proven advantageous as adjuvants, their mechanisms of action, including their ability to undergo structural modifications to achieve optimal immunogenicity, have been explored less thoroughly. In this work, we characterized the effects of two protein-based adjuvants (PBAs) on TLR4 signaling via the recruitment of MyD88 and TRIF. As models of TLR4-PBAs, we used hemocyanin from *Fissurella latimarginata* (FLH) and a recombinant surface immunogenic protein (rSIP) from *Streptococcus agalactiae*. We determined that rSIP and FLH are partial TLR4 agonists, and depending on the protein agonist used, TLR4 has a unique bias toward the TRIF or MyD88 pathway. Furthermore, when characterizing gene products with MyD88 and TRIF pathway-dependent expression, differences in TLR4-associated signaling were observed. rSIP and FLH require MyD88 and TRIF to activate nuclear factor kappa beta (NF-κB) and interferon regulatory factor (IRF). However, rSIP and FLH have a specific pattern of interleukin 6 (IL-6) and interferon gamma-induced protein 10 (IP-10) secretion associated with MyD88 and TRIF recruitment. Functionally, rSIP and FLH promote antigen cross-presentation in a manner dependent on TLR4, MyD88 and TRIF signaling. However, FLH activates a specific TRIF-dependent signaling pathway associated with cytokine expression and a pathway dependent on MyD88 and TRIF recruitment for antigen cross-presentation. Finally, this work supports the use of these TLR4-PBAs as clinically useful vaccine adjuvants that selectively activate TRIF- and MyD88-dependent signaling to drive safe innate immune responses and vigorous Th1 adaptive immune responses.

## 1 Introduction

In recent decades, TLR agonists have been investigated as possible vaccine adjuvants. Most compounds with adjuvant effects, such as lipopolysaccharide and oligonucleotides, are nonprotein microbial components. However, many studies have reported that TLR-dependent immunomodulation can be activated by numerous xenogeneic proteins in a thymus-dependent manner (1, 2). However, the potential role of these proteins as adjuvants requires a deeper understanding of their mechanisms of action to enable the creation of adjuvants with more powerful and more specific immunological effects. Among the many known TLR agonists, TLR4 ligand-based adjuvants are the most commonly used for developing commercial vaccines (3, 4). However, an improved understanding of TLR4 receptor−ligand interactions, signaling pathways, and biological/immunological mechanisms is needed to develop safe and potent vaccine formulations (5–7).

TLR4 is a transmembrane protein in leukocytes that belongs to the leucine-rich repeat family of proteins. It is activated by lipopolysaccharide (LPS), which triggers innate responses against gram-negative pathogens (8). Interaction of a TLR with its corresponding ligand agonist results in TLR dimerization, which triggers the recruitment of adapter proteins to the Toll IL-1 receptor (TIR) in the cytoplasmic domain of TLR4. This dimerization-based signaling process is an essential step in TLR4 signaling in which cytosolic TIR domains are activated to recruit adapter molecules, such as myeloid differentiation primary response factor 88 (MyD88), adapter-like MyD88 (MAL), the TIR domain-containing interferon-β inducer adapter (TRIF), and TRIF-related adapter molecule (TRAM), which then facilitate downstream signaling (9–12).

MyD88 signaling is associated with the rapid production of proinflammatory cytokines and innate immune responses to infectious threats (13, 14). In contrast, TRIF signaling is associated with processes that can promote adaptive immune responses essential for effective vaccination (10, 11, 15). Considering the roles of TLR4 agonists, most studies have used LPS-based ligands. However, a growing number of TLR4 protein agonists that could be used as vaccine adjuvants have been described (2, 16). Indeed, protein TLR4 agonists have several unique properties, including the ability to undergo structural modulation, optimal immunogenicity, and minimal toxicity (2, 16, 17). In this context, we analyzed two protein-based adjuvant agonists of TLR4 in terms of activation of the MyD88 and TRIF signaling pathways: one of bacterial origin, namely, the surface immunogenic protein (SIP) of Group *B Streptococcus* (GBS), and the other of molluskan origin, namely, hemocyanin from *Fissurella latimarginata* (FLH).

Gastropod hemocyanins are large metallo-glycoproteins of high molecular weight (approximately 8 to 13 MDa) that possess a complex quaternary structure and induce humoral and cell-mediated responses of the Th1 type in mammals, including humans. Due to this property, hemocyanins are widely used in biomedicine (17–22). In addition, different mollusk hemocyanins with immunological effects have also been characterized, such as FLH from *Fissurella latimarginata* (23–25). Studies performed in our laboratory showed that FLH has antitumor effects in murine melanoma and oral cancer models (20). In addition, FLH binds to TLR4 and induces the expression and secretion of Th1-type proinflammatory cytokines (17, 23, 25). A remarkable characteristic of hemocyanins is their carbohydrate content, which is fundamental to their structure and immunological efficacy (23, 26). Recent work showed that the enzymatic N-deglycosylation of FLH influences its immunogenic effects on macrophages (23), leading to a decrease in its binding to C-type lectin receptors, such as mannose receptor (MR), macrophage galactose lectin receptor (MGL), DC-specific intracellular adhesion molecule (ICAM)-grabbing nonintegrin (DC-SIGN), and TLR4 (17, 23, 25).

In contrast to hemocyanins, including the FLH used in this study, the recombinant surface immunogenic protein (rSIP) from Group B *Streptococcus* is a small protein. It was previously expressed in *Escherichia coli* and *Pichia pastoris* and had a molecular weight of 53 kDa with a β-folded structure and the ability to form dimers (27). This recombinant protein was analyzed as a vaccine against GBS in a preclinical trial. It was shown that this protein has excellent immunogenic capacity, as it binds TLR4 and induces a Th1-type response against GBS (28–31). Furthermore, given that SIP less complex structure than hemocyanins, it can undergo genetic fusion with other protein antigens to ensure joint antigen–adjuvant delivery (2). Previously, immunization with rSIP without adjuvant was shown to decrease GBS vaginal colonization and induce secretion of opsonizing antibodies evaluated by *in vitro* opsonophagocytosis (OPA) assays (30). Furthermore, rSIP was found to promote humoral immunity in a murine model using ovalbumin (OVA) as an antigen (27). Thus, considering that rSIP immunogenicity studies are in the advanced preclinical stage, this protein is a new candidate vaccine adjuvant.

In this work, we focused on characterizing two TLR4 protein agonists, their associations with MyD88 and TRIF recruitment and their contributions to antigen cross-presentation to CD8+ T lymphocytes. Since rSIP and FLH differ in origin, structure, and size, we hypothesized that MyD88 and TRIF recruitment is important for generating the TLR4-dependent Th1 effects of these protein-based adjuvants (PBAs). For this purpose, we studied the role of rSIP and FLH in the recruitment of MyD88 and TRIF in antigen-presenting cells (APCs) by characterizing molecular targets involved in TLR4 activation, as well as their adjuvant effects on antigen cross-presentation in bone marrow-derived dendritic cells (BM-DCs).

## 2 Materials and methods

### 2.1 Hemocyanin, rSIP, and ovalbumin antigen

*F. latimarginata* hemocyanin (FLH) was provided by Biosonda SA (Santiago, Chile). This protein was isolated and purified under sterile, pyrogen-free conditions in phosphate-buffered saline ([PBS] containing sodium phosphate 0.1 M NaCl), pH 7.2 (24), and Tris buffer for FLH containing 50 mM Tris, pH 7.4, 5 mM CaCl 2.5 mM MgCl_2_, and 0.15 mM NaCl (25). All chemicals were analytical reagent grade, and solutions were prepared with human irrigation water (Baxter Healthcare, Charlotte, NC, USA) and filtered through a 0.2 µm membrane filter (Millipore).

rSIP was obtained according to a procedure previously published by our group (27, 31). Briefly, rSIP was expressed in *E. coli* BL21 (DE3) and transformed into the plasmid pET21a::sip. Then, rSIP was expressed as a soluble protein and purified using nickel-nitrilotriacetic acid (NI-NTA) resin by low-pressure chromatography and high-performance liquid chromatography (HPLC) using a molecular exclusion column. The system consisted of a BioSep-SEC-s2000 300 x 21.2 mm Preparative Column 00H-2145-P0 (PHENOMENEX) and Smartline UV detector 2520 (Knauer, WissenschaftlicheGeräte GmbH, Germany). rSIP had a purity > 98%.

rSIP and FLH has endotoxin levels less than 0.5 EU/mL, which was determined using the ToxinSensor™ Chromogenic LAL Endotoxin Assay Kit. Additionally, protein concentrations were determined using the Pierce 660 nm Protein Assay Reagent (Thermo Scientific, Waltham, MA) according to the manufacturer’s instructions with a Pierce™ Bovine Serum Albumin Standard (Thermo Scientific).

Endotoxin-free OVA protein (Invivogen, cat. vac-stova) was used as the model antigen.

The stimuli and inhibitors used in this work did not induce cell toxicity in any cells used. Viability was determined with Trypan Blue and Annexin-V/propidium iodide (data not shown). Additionally, rSIP and FLH concentrations are reported as molar concentrations due to their significant differences in size and molar mass (rSIP ≈ 53 kDa; FLH ≈ 8,000 kDa).

### 2.2 Experimental animals

Mice of the wild-type C57BL/6 strain, C57BL/6-Tg (TcraTcrb)1100Mjb/J mice (OT-I), and B6.Cg-Tg(TcraTcrb)425Cbn/J (OT-II) mice were purchased from Jackson Laboratory. In addition, OT-I and OT-II were supplied by Fundación Ciencia & Vida (Chile). All mouse experiments followed international ethical standards and Chilean Animal Protection Law 20380 (2009). The Institutional Committee reviewed the experimental protocol in accordance with the Care and Use of Laboratory Animals of the Institute of Public Health of Chile, codes C110322-01 and C120421-01. The mice were housed in the Facility of the Laboratory Animal Maintenance and Experimentation Room (MEAL) of the Biotechnology Section of ISPCh. The mice were maintained following the regulations established by the Institutional Committee for the Use and Care of Animals of the laboratory.

### 2.3 Acquisition and culture of BM-DCs

BM-DCs were prepared using a modified procedure based on Lutz et al. (32). Briefly, bone marrow was extracted from the femurs and tibias of mice, washed with Hanks saline solution (HBSS), and cultured in BM-DC-specific medium containing Roswell Park Memorial Institute (RPMI-1640, Cytiva, cat. SH30027.02) supplemented with 10% inactivated fetal bovine serum (GIBCO, cat. 26140079), 2 mM L-glutamine, 1 mM sodium pyruvate, penicillin (50 U/ml), streptomycin (50 mg/ml), 50 mM β-mercaptoethanol, and 20 ng/ml granulocyte-macrophage colony-stimulating factor (GM-CSF; Peprotech, Cat. 315-03) to generate BM-DCs. Cells were seeded in a Petri dish at 2 x 10^6^/mL and incubated at 37°C. On days 3, 6, and 8, 10 ml of BM-DC medium was added to the cultures. On days 6 and 8, 10 ml of BM-DC medium was removed and replaced with fresh medium. On day 10, nonadherent BM-DCS were harvested. The BM-DCs were phenotypically characterized using flow cytometry (FACSVerse) after reaching > 85% expression of the phenotype CD11c+ CD11b+ MHCII+ CD86low CD80low CD4− CD8- B220− GR1−.

### 2.4 Cytokine secretion assay

The measurement of cytokines in the supernatant of BM-DCs cultured with FLH and rSIP was carried out according to Kolb et al. (12). BM-DCs (1 x 10^5^ per well) were incubated in flat-bottom 96-well plates for 2 h at 37°C before TLR4 protein agonists (FLH 120 nM and rSIP 40 nM) or PBS (vehicle control) was added. TRIF and MyD88 inhibition experiments were performed as described by Chen et al. (33) in which 75 µM Pepinh-TRIF (Humimmu LLC), 75 µM Pepinh-Control (Negative Control, Humimmu LLC), 75 µM MyD88 peptide control (Novus Biological), and 75 µM antennapedia control peptide (Novus Biological, negative control) were added 18 h before the addition of FLH and rSIP.

For the TLR4 signaling inhibitor, TAK242 was used according to the supplier’s recommendations (34). TAK 242 (10 μg/mL, Invivogen) was added 2 h before the addition of FLH and rSIP. After 18 hours of stimulation with FLH and rSIP at 37°C, the supernatants were collected. The concentrations of IL-6 and IP-10 were measured using a commercial enzyme-linked immunosorbent assay (ELISA) kit (Mouse IL-6 ELISA Kit: BMS603-2; Mouse IP-10 ELISA Kit: BMS6018, Invitrogen) according to the manufacturer’s instructions and with all necessary controls. The sensitivity of both ELISA kits was 6.5 pg/mL. The assay range for the IL-6 ELISA kit was 31.3-20,000 pg/mL. The assay range for the IP-10 ELISA kit was 7.8-500 pg/mL.

The inhibitors did not induce cell toxicity in the cells used in these assays (data not shown).

### 2.5 Determination of gene expression by real-time reverse-transcriptase polymerase chain reaction (RT−qPCR)

The measurement of mRNA from BM-DCs was carried out according to Kolb et al. (12). BM-DCs (1x10^5^ per well) were incubated in flat-bottom 96-well plates for 2 hours at 37°C before TLR4 protein agonists or PBS (vehicle control) were added. MyD88 and TRIF pathway inhibitors were used in the manner described above. Once stimulated, the cells were washed with cold HBSS. Cell lysis and total RNA isolation were performed with NucliSENS® easyMAG equipment; Biomérieux and complementary DNA (cDNA) were synthesized with SuperScript™ III Reverse Transcriptase (Invitrogen). Assays were performed using 1 μl of gDNA template and a Stratagene Mx3000P thermocycler (Agilent Technologies). Increases in mRNA abundance in treated cells relative to control cells were calculated using the 2−ΔΔCt method and normalized to β-actin mRNA. The sequences of primers and probes used for detecting mRNAs can be found in **Supplemental Table 1**.

### 2.6 THP-1 Dual cell culture

THP-1 Dual cells (InvivoGen, thpd-nfis), TRIF KO Dual Reporter THP1 Cells (InvivoGen, thpd-kotrif), THP1-Dual™ KO-MyD cells (InvivoGen, thpd-komyd), and TLR4 KO Dual Reporter THP- 1 Cells (InvivoGen thpd-koTLR4) were cultured in RPMI-1640 medium supplemented with 10% fetal bovine serum (Gibco), L-glutamine, 100 U/ml penicillin, 100 mg/ml streptomycin (Gibco), 100 mg/mL normocin (InvivoGen, ant-nr-1), 100 mg/mL zeocin (InvivoGen, ant-zn-1), and 10 mg/mL blasticidin (InvivoGen, ant-bl-1). Dual THP1 cells were incubated at 37°C and 5% CO_2_.

THP1-Dual™ KO-TLR4, THP1-Dual™ KO-TRIF, and THP1-Dual™ KO-MyD88 cells were generated from THP1 Dual cells™ via knockout (KO) of TLR4, TRIF and MyD88 (InvivoGen). Previously, these cells were validated to characterize the functionality of SEAP and LUCIA expression. The activation of NF-kB and IRF was previously analyzed using a NOD1 agonist and TLR3 agonist. The NOD1 ligand generated an increase in SEAP in all cell lines. For LUCIA, all lines showed activation of IRF in the presence of the TLR3 agonist (data not shown).

### 2.7 SEAP and LUCIA assays in THP-1 and Hek-blue cells

THP-1 cells were seeded in 96-well plates (Corning Costar) at a density of 100,000 cells per well. First, cells were stimulated for 18 h with rSIP, FLH, LPS, a NOD 1 ligand (positive control for NF-kB; C12-iE-DAP, InvivoGen), and a PRR agonist (positive control for IRF; Poly (dA:dT)/LyoVec™, InvivoGen). The supernatant was then subjected to a colorimetric enzyme assay to measure alkaline phosphatase (AP) activity using the commercial QUANTI-Blue™ solution (InvivoGen). The supernatant was then incubated at 37°C for 3 h, and the optical density was read at 650 nm in an Epoch 2 reader (BioTek). On the other hand, luciferase activity (LUCIA) was measured using the commercial solution QUANTI-Luc ™ (InvivoGen), which has a coelenterazine substrate and stabilizing agents for the luciferase reaction. The light signal produced was then quantified using a Berthold luminometer (Model LB9515), and the signal was expressed as relative light units (RLUs).

Hek-Blue cells (hkb-mtlr4 and hkb-htlr4, InvivoGen) express SEAP under the control of promoters containing binding elements for the NF-κB transcription factor (35). Hek-Blue cells were seeded in 96-well plates (Corning Costar) at a density of 25,000 cells per well in HEK-Blue™ Detection medium (InvivoGen). Then, the cells were stimulated for 48 h with rSIP, FLH, and LPS, and SEAP was quantified using an Epoch 2 reader (Biotek).

### 2.8 Exogenous antigen presentation assays in an *in vitro* model

The presentation of exogenous antigens in an *in vitro* model was evaluated as described by Alloatti et al. (36). BM-DCs (1 x 10^5^ per well) were incubated in flat-bottom 96-well plates for 2 h at 37°C before OVA and TLR4 agonists were added. The BM-DCs were incubated with OVA, OVA + FLH, OVA +SIP, or PBS. After 24, 48 and 72 h, the BM-DCs were washed three times with a 0.1% (vol/vol) PBS/BSA solution and then labeled with the 25-D1.16 antibody that detects peptide–major histocompatibility class (MHC)-I (SIINFEKL: MHC-I) complexes (37).

### 2.9 Antigen cross-presentation assay

The antigen cross-presentation assay was adopted and modified from Alloati et al. (36). Following TLR4-induced maturation of DCs, antigen cross-presentation is first enhanced and then modulated downstream of antigen internalization and cytosolic delivery (36, 38). It was previously reported that antigen cross-presentation capacity increased in the initial hours after TLR4 activation (39). To evaluate antigen cross-presentation, BM-DCs were seeded at 40 x 10^3^ cells per well in a 96-well plate and then pulsed with the antigens at different concentrations for 3 h. At the end of the pulsing period, the cells were washed three times to remove excess antigen and cocultured with 1 x 10^5^ CD8 T cells purified from the spleens of OT-I mice using the MojoSort™ Mouse CD8 T-Cell Isolation Kit according to the manufacturer’s protocol. CD8 T cells from OT-I mice were labeled with CellTrace Violet™ (Molecular Probes™, ThermoFisher Scientific). Three days later, the proliferation of CD8 T cells was measured using flow cytometry. As a proliferation control, OT-I lymphocytes were cocultured with BM-DCs pulsed with the ovalbumin peptide SIINFEKL (Invivogen).

For MyD88 and TRIF inhibition, the peptides were added to BM-DCs 18 hours before pulsing with antigen and adjuvant. TAK-242 (10 µg/mL, Invivogen) was added 2 hours before washing and pulsing with more antigen adjuvant. The BM-DCs were cocultured with 30x10^4^ CD8 T cells, and cell proliferation was characterized as described above.

A similar approach was used to assess the effect of the proteasomal, vacuolar, and endoplasmic reticulum trafficking processes using several inhibitors: (A) Brefeldin A at 1 µM, (B) Epoxomicin at 5 nM, (C) Leupeptin at 10 µM, (D) Pepstatin A at 40 nM, (E) MG132 at 4 nM, (F) Bafilomycin at 10 nM, and (G) Simvastatin at 10 nM. All inhibitors were obtained from Enzo. The inhibitors were added to BM-DCs 1 h before washing, pulsing with antigen and adjuvant, and coculture with 30 x 10^4^ CD8 T cells (OT-I) as described above.

### 2.10 Classical antigen presentation assay

The classical presentation of antigens was evaluated similarly to antigen cross-presentation, with minor modifications. BM-DCs were seeded at 40 x 10^3^ cells per well in a 96-well plate and then pulsed with the antigens at different concentrations for 18 h. At the end of the pulsing period, the cells were washed three times to remove excess antigen and cocultured with 1 x 10^5^ CD4 T cells purified from the spleens of OT-II mice using the MojoSort™ Mouse CD4 T-Cell Isolation Kit according to the manufacturer’s protocol. CD4 T cells from OT-II mice were labeled with CellTrace Violet™ (Molecular Probes™, ThermoFisher Scientific). Four days later, the proliferation of CD4 T cells was measured using flow cytometry. As a proliferation control, OT-II lymphocytes were cocultured with BM-DCs pulsed with the OVA peptide ISQAVHAAHAEINEAGR (InvivoGen).

### 2.11 Immunoblotting

Immunoblotting was performed according to Jiménez et al., with modifications (17). BM-DCs (4 × 10^6^) were incubated for 2 h at 37°C in polystyrene tubes and were then exposed to rSIP or FLH. The cells were lysed at the indicated time points using RIPA lysis buffer supplemented with protease inhibitor cocktail (5 mg/mL; Roche). Proteins were separated in polyacrylamide gels (SDS−PAGE, 10-15%), electrotransferred to nitrocellulose membranes, and blocked with 5% bovine serum albumin (BSA) in 0.1% (v/v) TBS-Tween 20 (TBST). Primary antibodies were dissolved in 5% BSA and incubated with the blocked membranes overnight at 4°C. After exposure of the membranes to horseradish peroxidase-conjugated anti-rabbit secondary antibodies for 1 h in BSA, bands were visualized using the Odyssey system (Li-Cor Bioscience) detection system, and band intensities were analyzed with LI-COR Image Studio Software. SuperSignal West Femto Maximum Sensitivity Substrate (Thermo Fisher) and EZ-ECL (Biological Industries) were the chemiluminescent substrates used for developing the Western blots. The antibodies used for immunoblotting were a recombinant anti-IRF3 antibody [EPR2418Y] (ab68481), goat anti-rabbit IgG H&L (HRP) (Abcam, ab205718) and phospho-IRF-3 (Ser396) (4D4G) rabbit mAb #4947 (Cell Signaling Technology, Danvers, USA).

### 2.12 Statistical analysis and determination of log (EC_50_) values

The log half maximal effective concentration (EC_50_) values for each agonist-induced response were calculated according to Ehlert et al. (40), generating nonlinear fits for four parameters defined as the baseline response line (Bottom), maximum response (Top), slope of the curve (HillSlope), and concentration of protein agonist that elicited a median response between the baseline and upper response (EC_50_). Dose−response data were analyzed using GraphPad Prism software with the following equation: Y=Bottom + (X^Hillslope)*(Top-Bottom)/(X^HillSlope + EC50^HillSlope).

Differences between log (EC_50_) values were analyzed using GraphPad Prism 9 software by applying a two-tailed t test (for comparisons between two sets of proteins). In addition, the statistical significance of differences in inhibition by TAK-242, MyD88, and TRIF inhibitors was evaluated with the one-tailed Mann–Whitney U test or the Kruskal−Wallis test followed by *post hoc* tests for multiple comparisons.

## 3 Results

### 3.1 FLH and rSIP differ in their potency as TLR4 agonists

**Figure 1:**
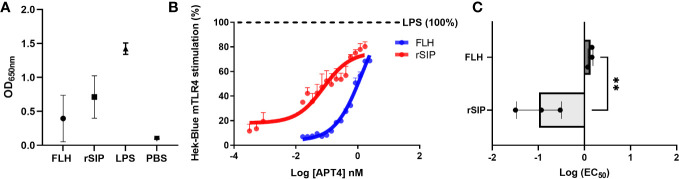
Partial agonism of mTLR4 by protein-based adjuvants. **(A, B)** Raw data and determination of the concentrations of protein agonists needed to activate mTLR4. The HEK-Blue-mTLR4 reporter cell line was exposed to different concentrations of rSIP and FLH. Dose−response curves were generated for cells exposed to a maximum concentration of 2540 nM rSIP or FLH for 48 h. Data show normalized HEK-Blue mTLR4 cell responses considering treatment with lipopolysaccharide (LPS) as 100% stimulation; 100% = maximum dose plateau of the LPS agonist. **(C)** Comparison of the log EC_50_ values of protein agonists in the activation of mTLR4. Log (EC_50_) values for rSIP and FLH were determined according to the relative abundance of soluble alkaline phosphatase (AP) secreted by Hek-Blue-mTLR4 cells. Individual log (EC_50_) values and mean values from three independent experiments are shown. The statistical significance of differences was analyzed using an unpaired t test (**p < 0.01).

Understanding the modulation of TLR4 agonist-mediated signaling is pivotal for deciphering the immune mechanisms to develop vaccine adjuvants (41). To gain insight into the mechanism underlying TLR4 protein agonists, we performed dose−response analysis of HEK-blue reporter cells expressing either mouse TLR4 (mTLR4) or human TLR4 (hTLR4). Analyses were performed to evaluate LPS stimulation, including a full TLR4 agonist and PBS as a negative control. In the raw data, rSIP induced stronger stimulation than FLH according to the dose−response curve (**Figure 1A**). rSIP and FLH were partial agonists of mTLR4 and reached a maximum activation value of approximately 80% compared to the full agonist LPS (4), as shown in **Figure 1B**. Therefore, the half-maximal effective concentrations of the two proteins were compared to determine whether a difference in the immunological potency of these partial agonists could be observed. The findings showed that rSIP had a lower EC_50_ than FLH and a mean log (EC_50_) value of –1, compared to FLH, which had a mean log (EC_50_) value of 0.1; therefore, these agonists stimulated mTLR4 at lower protein concentrations (**Figure 1C**).

Different mTLR4 agonists used to characterize preclinical animal models differ significantly from those observed in human cell systems (42). In this context, the agonist effects of rSIP and FLH on Hek-Blue hTLR4 cells was characterized (**Supplemental Figure 1A**). Compared to LPS (100% activation), rSIP was found to be a partial agonist of hTLR4, while FLH acted as a lower efficacy agonist of hTLR4. This conclusion was confirmed by the observation that LPS activated HEK-Blue-TLR4 cells at 18 h post-stimulation, while rSIP and FLH stimulated the cells at 48 h post-stimulation, with rSIP reaching approximately 90% activation and FLH reaching 25% activation. The above result suggests that TLR4-PBAs are ligands of hTLR4, with partial and weak partial agonist effects for rSIP and FLH, respectively. Although a difference in the potency of hTLR4 activation was found, these agonists presented similar log(EC_50_) values (**Supplemental Figure 1B**). HEK-Blue Null1-v cells, which do not express mTLR4 or hTLR4, were used as a negative control for the cell line and were not activated in the presence of FLH or rSIP (data not shown). Notably, both rSIP and FLH were able to activate NF-κB in the HEK-Blue-TLR4 cell line. Furthermore, both proteins were able to activate IRF3 in BM-DCs (**Supplemental Figure 1C**). Our results show the specificity of mTLR4 for the protein ligands described above, and we propose that the structural differences between these ligands, i.e., FLH is very large and glycosylated, and rSIP is small and not glycosylated, will allow them to serve as new models to study their contributions to MyD88 and TRIF signaling.

### 3.2 TLR4-PBAs show no bias toward MyD88 or TRIF signaling at minimal activation doses

**Figure 2:**
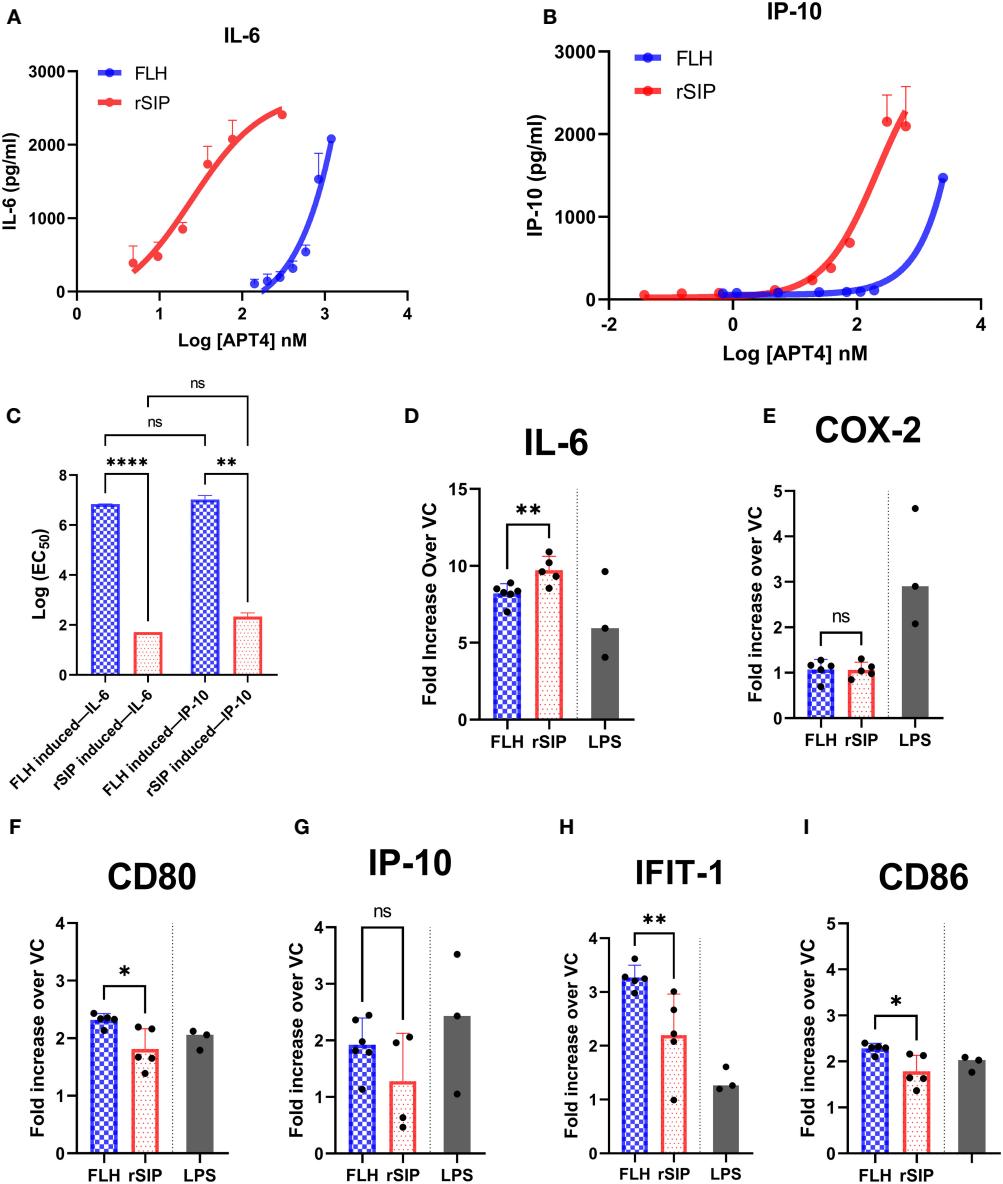
FLH and rSIP induce differential expression patterns for molecules associated with the MyD88 and TRIF pathways. Bone marrow dendritic cells (BM-DCs) from C57BL/6 mice were treated with the indicated concentrations of rSIP and FLH. **(A, B)** Analysis of IL-6 and IP-10. After 18 h, the concentrations of IL-6 and IP-10 were determined using enzyme-linked immunosorbent assay (ELISA). Data are expressed as the means ± standard deviations (SD) of three independent experiments. **(C)** Log (EC_50_) comparison. The log (EC_50_) values of the indicated rSIP- and FLH-stimulated changes in interleukin 6/(IL-6/IP-10) expression from experiments A and B were compared. Individual log (EC_50_) values are shown. Values are the means of three independent experiments. Statistical differences were analyzed for the data in **(C)** using the Mann–Whitney U test (**p<0.01; ****p<0.0001; ns, statistically not significant). **(D–I)** Analysis of MyD88– and TRIF–codependent expression of target genes. Wild-type BM-DCs were stimulated with rSIP (40 nM) and FLH (120 nM) for 4 h. The mRNA abundances of **(D)** IL-6, **(E)** cyclooxygenase 2 (COX-2), **(F)** cluster of differentiation 80 (CD80), **(G)** IP-10, **(H)** IFIT-1, and **(I)** CD86 were analyzed by RT−qPCR. LPS-stimulated BM-DCs were used as a positive control for each of the mRNAs analyzed. Data are expressed as the mean fold increase in mRNA abundance in cells stimulated with protein adjuvants compared to cells treated with PBS. Each dot represents an independent experiment. Data are the means ± SDs of 4 or 5 independent experiments. Statistical significance was determined using the Mann–Whitney U test (*p<0.05; **p<0.01; ns, statistically not significant).

To determine whether rSIP and FLH are agonists biased toward the TRIF or MyD88 pathway, BM-DCs were activated with an extensive dilution series of rSIP and FLH. The potencies of these agonists in activating a panel of TRIF-dependent and MyD88- and TRIF-codependent proteins were measured by calculating the log (EC_50_) values. We evaluated IP-10 as a representative TRIF-dependent protein and IL-6 as a MyD88- and TRIF-codependent cytokine (12). As expected, based on the log (EC_50_) value, rSIP was more active than FLH in inducing the expression of TRIF-dependent and TRIF-codependent proteins (MyD88 and TRIF) (**Figures 2A–C**). rSIP produced mean log (EC_50_) values of 1.8 and 2.1 for IL-6 and IP-10, respectively, while FLH produced mean log (EC_50_) values of 6.8 and 7.1 for IL-6 and IP-10, respectively. However, no log (EC_50_) difference for the comparison of IL-6 and IP-10 expression between rSIP and FLH was found (**Figure 2C**). Taken together, these results suggest that at minimal activation doses, rSIP has a lower Log (EC_50_) than FLH, which is consistent with the results presented in **Figure 1**. No differences in the preferential activation pathway were found when the log (EC_50_) values for IL-6 and IP-10 were compared. FLH generated the same effect. Therefore, FLH and rSIP induce MyD88- and TRIF-codependent pathways, as reflected by IL-6 and IP-10 expression.

Furthermore, we aimed to characterize other molecules associated with the recruitment of MyD88 and TRIF. The MyD88- and TRIF-codependent pathway is characterized by the gene expression of IL-6, Cox-2, and cluster of differentiation 80 (CD80). Expression of the IP-10, IFIT1, and CD86 mRNAs is associated with the TRIF-dependent pathway. BM-DCs were stimulated with EC_50_ doses of rSIP and FLH, and the mRNAs of IL-6, cyclooxygenase (COX-2), CD80, IP-10, interferon-induce protein with tetratricopeptide repeats (IFIT-1), and CD86 were evaluated (**Figures 2D–I**). RT−qPCR revealed that FLH induced higher expression of CD80, IFIT-1, and CD86 than rSIP. On the other hand, rSIP induced higher expression of IL-6 than did FLH. In contrast, COX-2 and IP-10 expression showed no variations associated with either rSIP or FLH. This result suggests that different profiles associated with the recruitment of MyD88 and TRIF could occur for rSIP and FLH at their respective EC_50_ values. This fine signaling regulation mediated by TLR4 implies some cross-regulation of these pathways.

### 3.3 The regulation of FLH– and rSIP−TLR4 activation is associated with MyD88- and TRIF-dependent genes

**Figure 3:**
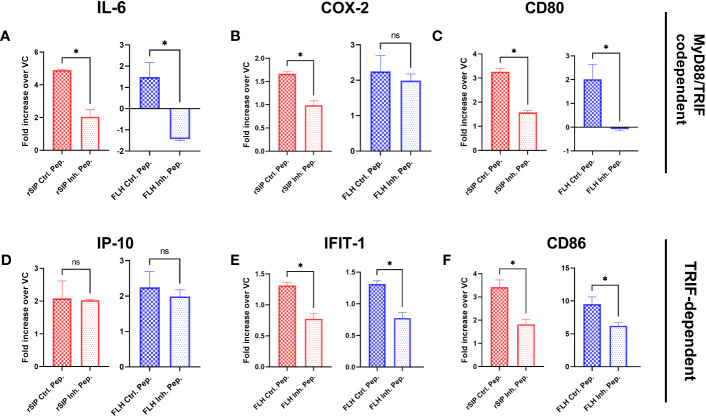
MyD88 is required for the activation of signaling pathways by protein-based adjuvants. Wild-type BM-DCs were pretreated with peptide inhibitors of MyD88 or a peptide control and stimulated with rSIP (40 nM) and FLH (120 nM) for 4 h. **(A–F)** Quantification of RNA. The mRNA abundances of **(A)** IL-6, **(B)** COX-2, **(C)** CD80, **(D)** IP-10, **(E)** IFIT-1, and **(F)** CD86 were analyzed by reverse-transcriptase polymerase chain reaction (RT−qPCR). Data are the mean fold increase in mRNA abundance in cells stimulated with protein adjuvants compared to cells treated with PBS (vehicle control, VC) and averaged from three independent experiments. The activation of TLR4 by rSIP is dependent on MyD88 recruitment for the activation of **(A)** IL-6, **(B)** COX-2, **(C)** CD80, **(E)** IFIT-1, and **(F)** CD86 expression. On the other hand, the activation of TLR4 by FLH is dependent on the recruitment of MyD88 for the activation of **(A)** IL-6, **(C)** CD80, **(E)** IFIT-1, and **(F)** CD86 expression, but the activation of **(D)** IP-10 expression was not dependent on MyD88 after activation by FLH and rSIP. Data are represented as the means ± SDs of three independent experiments. Statistical significance was determined using the Mann–Whitney U test (*p<0.05; ns: not statistically significant).

Since a preference for the MyD88 or TRIF pathway was not observed, we decided to further examine the effects of these pathways on the immune response induced by both adjuvant proteins. To characterize the contribution of MyD88 to TLR4 activation by rSIP and FLH, BM-DCs were pretreated for 18 h with the MyD88 inhibitor, an inhibitory peptide that blocks MyD88 signaling, or a control peptide prior to stimulation of BM-DCs for 4 h, and RT−qPCR was performed. Inhibition of MyD88 decreased IL-6, CD80, CD86, and IFIT1 transcript levels but not COX-2 transcript levels when BM-DCs were stimulated with FLH (**Figure 3**). For rSIP, inhibition of MyD88 induced a decrease in IL-6, CD80, CD86, IFIT1, and COX-2 transcript levels (**Figure 3**). On the other hand, no variation in the number of IP-10 transcript levels upon stimulation with rSIP and FLH was found, suggesting that the TLR4 agonistic effects of rSIP and FLH are MyD88 dependent.

**Figure 4:**
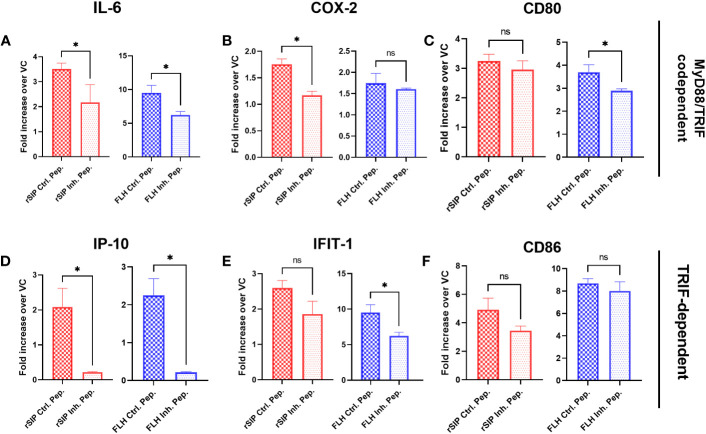
Activation of IP-10 and IL-6 by TLR4-PBAs is dependent on TRIF. Wild-type BM-DCs were pretreated with the TRIF peptide inhibitor or a peptide control and stimulated with rSIP (40 nM) and FLH (120 nM) for 4 h. **(A–F)** Quantification of RNA. The mRNA abundances of **(A)** IL-6, **(B)** cyclooxygenase 2 (COX-2), **(C)** cluster of differentiation 80 (CD80), **(D)** IP-10, **(E)** interferon-induced protein with tetratricopeptide repeat 1 (IFIT-1), and **(F)** CD86 were analyzed by RT−qPCR. Data are expressed as the mean fold increase in mRNA abundance in cells stimulated with the protein adjuvants compared to cells treated with PBS (vehicle control, VC) and averaged from three independent experiments. The activation of TLR4 by rSIP is dependent on the recruitment of TRIF for the activation of **(A)** IL-6, **(B)** COX-2 and **(D)** IP-10 expression. In contrast, the activation of FLH depends on the recruitment of TRIF to activate **(A)** IL-6, **(C)** CD80, **(D)** IP-10, and **(E)** IFIT-1 expression. Data are the means ± SDs of three independent experiments. Statistical significance was determined using the Mann–Whitney U test (*p<0.05; ns, not statistically significant).

Similar to the approach used for MyD88 inhibition, we decided to further examine the effects of TRIF on the immune response induced by the rSIP and FLH proteins. The BM-DCs were pretreated for 18 h with Pepinh-TRIF, an inhibitory peptide that blocks TRIF signaling, or Pepinh-Control, a control peptide, and then treated with rSIP and FLH. After the cells were stimulated for 4 h, RT−qPCR was performed. TRIF inhibition decreased the transcription of IL-6, COX-2, and IP-10 after stimulation with rSIP (**Figure 4**). Regarding FLH, TRIF inhibition decreased IL-6, CD80, IFIT1, and IP-10 expression; however, no effects on CD86 transcript levels in response rSIP and FLH were found. Therefore, TRIF is required for the activation of IL-6 and IP-10 expression by FLH and rSIP (**Figures 4A, D**). These results indicate that TLR4, MyD88, and TRIF are important for the signaling patterns associated with TLR4-PBAs in BM-DCs.

### 3.4 rSIP activates MyD88− and TRIF− dependent proteins, while FLH activates TRIF− dependent proteins

**Figure 5:**
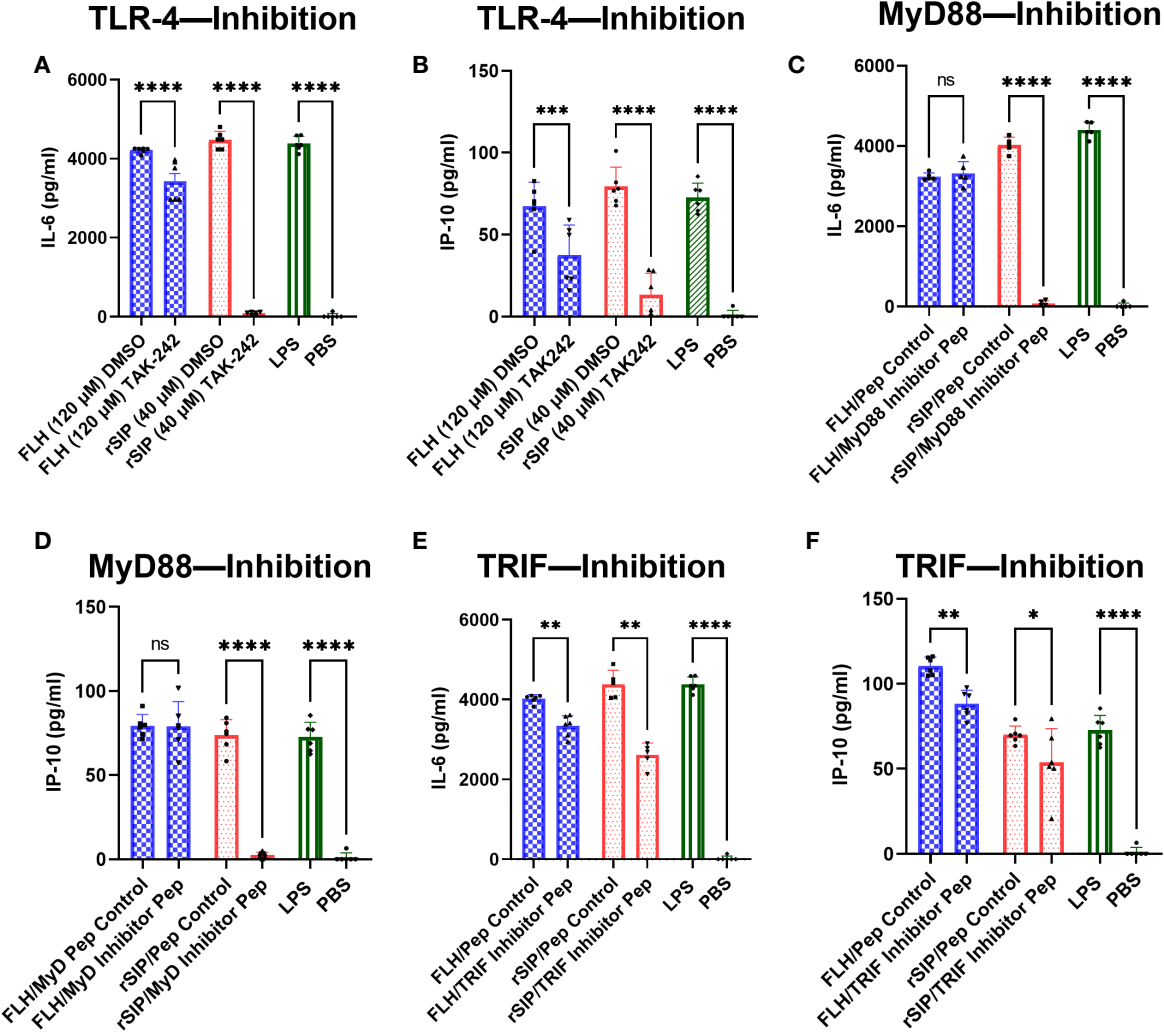
rSIP and FLH differ in signaling due to the recruitment of MyD88 and TRIF. Wild-type BM-DCs were pretreated with **(A, B)** TLR4 inhibitors and peptide inhibitors of **(E, F)** TRIF and **(C, D)** MyD88 and stimulated with rSIP (40 nM) and FLH (120 nM) for 18 h. Next, IL-6 and IP-10 were analyzed using ELISA. Assays were validated using LPS as a positive control and PBS as a negative control. Each dot represents an independent experiment. Data are the means ± SDs of six independent experiments. Statistical significance was determined using repeated measures one-way analysis of variance (ANOVA) and the *post hoc* Sidak test (*p<0.05; **p<0.01; ***p<0.001; ****p<0.0001; ns, not statistically significant).

After establishing an association between TLR4-PBAs and genes with MyD88- and TRIF-dependent expression at minimal activation concentrations, no preference for the TRIF and MyD88 pathways was observed. Therefore, we next sought to address whether inhibition of MyD88 and TRIF influences IL-6 and IP-10 secretion by BM-DCs. First, we characterized the effects of TLR4 on cytokine secretion. For this purpose, the cells were treated for 2 h with TAK-242 or dimethyl sulfoxide (DMSO) as the negative control. TAK-242 induced complete and partial inhibition of the IL-6 and IP-10 secretion induced by both rSIP and FLH, respectively (**Figures 5A, B**). These results suggest that the effect of rSIP and FLH on cytokine secretion by BM-DCS is dependent on TLR4 in BM-DCs. This effect was also reflected by the expression of CD86 by flow cytometry, which was dependent on TLR4 (data not shown).

To characterize the effect of MyD88, we used the methods described above, and we treated BM-DCs for 18 h with a MyD88 inhibitor peptide and then pulsed them with rSIP and FLH. Inhibition of MyD88 caused dramatic inhibition of IL-6 and IP-10 expression in rSIP-stimulated BM-DCs (**Figures 5C, D**). Conversely, IL-6 and IP-10 production induced by FLH was not affected by inhibition of MyD88 recruitment. Next, to characterize the effect of TRIF, we used peptides that inhibit the recruitment of TRIF (Pepinh-TRIF) in BM-DCs. As with the MyD88 inhibition methods, we pretreated cells with the TRIF-inhibiting peptide for 18 h and pulsed them with rSIP and FLH. TRIF inhibition, how, had a less dramatic but significant effect on the expression of IL-6 and IP10 in BM-DCs stimulated with rSIP and FLH (**Figures 5E, F**). These data suggest differential regulation of TLR4 signaling in terms of the recruitment MyD88 and TRIF by rSIP and FLH. They also highlight a slight difference in that MyD88 is important for rSIP-induced IL-6 and IP-10 secretion, whereas TRIF is essential for FLH-induced IL-6 and IP-10 secretion.

### 3.5 MyD88 and TRIF are required for NF-κB- and IRF-associated signaling during TLR4 activation by FLH and rSIP

**Figure 6:**
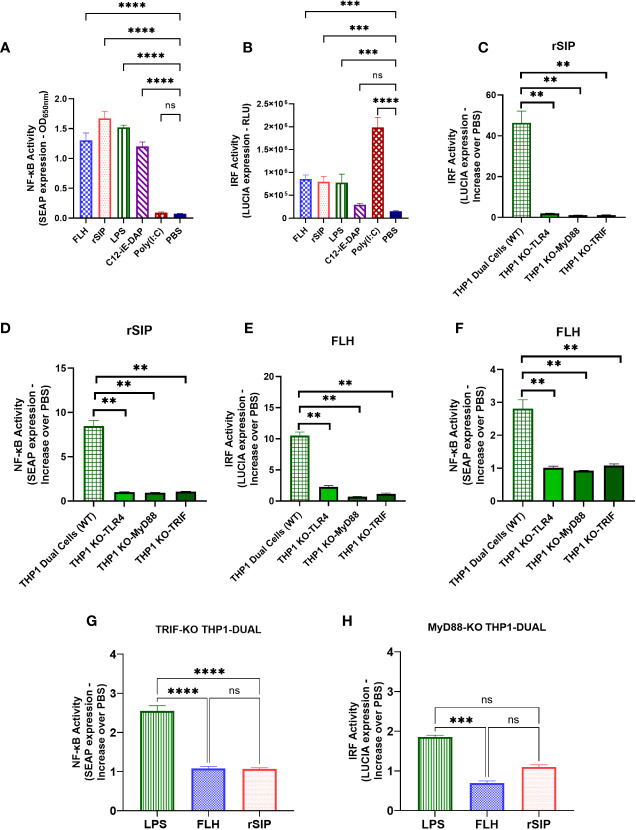
MyD88 and TRIF are required for NF-κB- and IRF-associated signaling after TLR4-PBA activation. THP1 Dual (wild-type) cell lines were treated with rSIP (0.02 µM) and FLH (2.45 µM) for 18 h. **(A, B).** The activity of **(A)** secreted alkaline phosphatase (SEAP) and **(B)** luciferase (LUCIA) was characterized after stimulation with rSIP, FLH, LPS (10 µg/ml), C12-iE-DAP (1 μg/ml; a nucleotide-binding oligomerization domain-containing protein 1 [NOD1] ligand), and poly(I:C) (1 μg/ml; a TLR3 ligand). The data are the average of the OD at 600 nm, and the relative light units (RLUs) are the average of three independent experiments. Statistical significance was determined by one-way analysis of variance (ANOVA) compared to the PBS control (***p<0.001; ****p<0.0001; ns, statistically not significant). **(C–G)**. The THP1 Dual (wild-type), MyD88-KO, TLR4-KO, and TRIF-KO cell lines were treated with rSIP and FLH for 18 h. SEAP activity was characterized after stimulation with **(C)** rSIP and **(E)** FLH in the THP1 Dual (wild-type), MyD88-KO, TLR4-KO, and TRIF-KO cell lines. Data are the average increase in SEAP induction compared to the negative control (PBS) and are averaged from three independent experiments. LUCIA activity in response to **(D)** rSIP and **(F)** FLH was then characterized in THP1 Dual (wild-type), MyD88-KO, TLR4-KO, and TRIF-KO cells. Data are the average increase in LUCIA induction compared to the negative control (PBS) and are averages of three independent experiments. Statistical significance was determined using one-way ANOVA and the Mann−Whitney U test (**p < 0.01). **(G, H).** The THP1-Dual TRIF-KO cell line and the THP1-Dual MyD-KO cell line were pulsed with rSIP (0.02 µM), FLH (2.45 µM), LPS (10 µg/ml), and PBS for 18 h. Then, SEAP activity and IRF activity were characterized. Data are expressed as the average of the increase compared to the negative control (PBS) for **(G)** SEAP induction in the TRIF-KO THP1 cell line and **(H)** LUCIA luciferase induction in the MyD88-KO THP1 cell line. The results are from three independent experiments. Statistical significance was determined by one-way analysis of variance (ANOVA) with Tukey’s multiple comparisons tests (***p<0.001; ****p<0.0001; ns, statistically not significant).

To obtain more information in a human model, we used THP1-Dual™ cells derived from the human THP-1 monocyte cell line to characterize the NF-kB and IRF pathways. These cells show stable integration of two inducible reporter constructs that allow the concurrent study of the NF-κB pathway by monitoring the activity of SEAP and the IRF pathway by assessing the activity of a secreted luciferase (LUCIA). Consistent with our previous data, the results showed that the two model proteins significantly induced the secretion of SEAP and LUCIA in the PBS control group (**Figures 6A, B**). Additionally, LPS was analyzed in the assay and stimulated both NF-kB and IRF at levels similar to those seen for our proteins. In addition, nucleotide-binding oligomerization domain-containing protein 1 (NOD1/C12-iE-DAP) and TLR3 [poly (I:C)] agonists were used as positive controls for the secretion of SEAP and LUCIA, respectively. The data suggest that protein agonists activate the NF-kB and IRF pathways.

To characterize the effects of TLR4 protein agonists on TRIF and MyD88 recruitment, THP1-Dual™(WT), KO-TRIF, KO-MyD88, and KO-TLR4 cells were pulsed for 18 h with rSIP and FLH at 0.02 µM and 2.45 µM, respectively. These concentrations are the minimum concentrations needed to achieve stimulation of the THP1 line via the NF-kB and IRF pathways. Then, the supernatants were used to evaluate the levels of SEAP associated with activation of the NF-κB pathway (**Figures 6C, D**) and the levels of LUCIA (**Figures 6E, F**) associated with activation of the IRF pathway. SEAP induction in response to rSIP and FLH were abolished in the THP1 Dual KO-TRIF, KO-MyD88, and KO-TLR4 cells, with levels approximately 9- and 3-fold lower than those in the WT control, respectively. Moreover, LUCIA signals in response to rSIP and FLH were abolished in the THP1 Dual KO-TRIF, KO-MyD88, and KO-TLR4 cells, with levels approximately 40-fold and 10-fold lower than those of the WT control, respectively.

Notably, LPS can activate the TRIF-independent NF-κB pathway and MyD88-independent IRF pathway (38, 43, 44). In this context, the activation of NF-κB and IRF was compared in the THP1-Dual KO-TRIF and THP1-Dual KO-MyD88 cell lines, and it was observed that the rSIP and FLH agonist proteins activate the NF-κB and IRF pathways (**Figures 6G, H**). LPS led to a 2.5-fold increase in SEAP levels compared to the control in the TRIF-KO THP1 cell line, while rSIP and FLH induced only a 1-fold increase compared to the control. The similar result obtained for IRF indicated that LPS activated the MyD88-KO THP1 cell line, with a 1.9-fold increase compared to the control, while FLH and rSIP caused only 1- and 0.8-fold activation, respectively, compared to the control. These results suggest that TLR4-PBAs are equally affected by the MyD88 and TRIF pathways and that NF-κB and IRF are essential for rSIP and FLH signaling.

### 3.6 rSIP and FLH promote antigen cross-presentation by recruiting MyD88- and TRIF-dependent proteins

**Figure 7:**
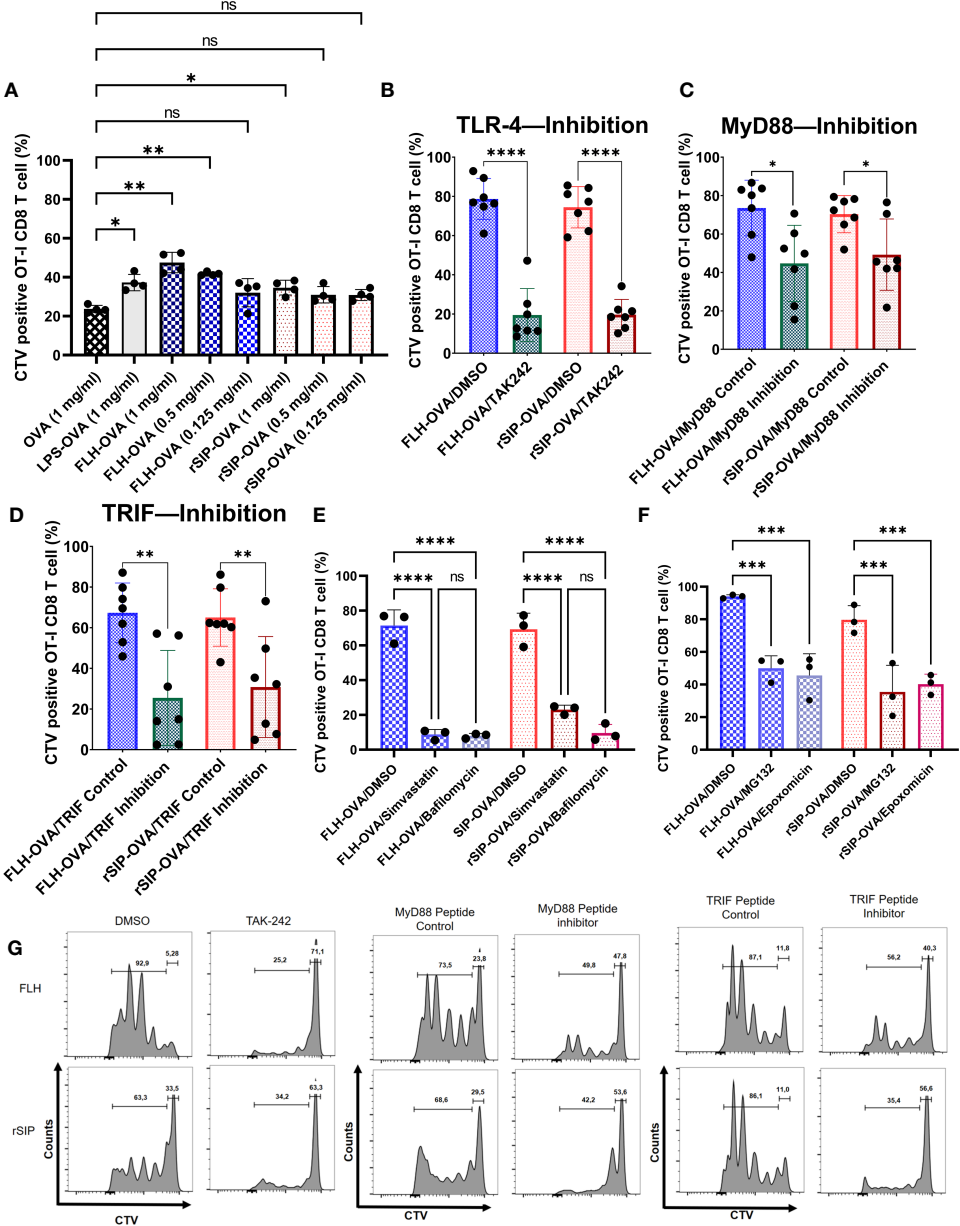
rSIP and FLH induce antigen cross-presentation dependent on MyD88 and TRIF recruitment. **(A)** Proliferation induced by OVA. BM-DCs were stimulated for 3 h with rSIP, FLH, and coadministered increasing concentrations of OVA (1 mg/mL, 0.5 mg/mL, and 0.125 mg/mL). Naïve OT-I CD8 T-cell (1 x 10^5^ cells) proliferation was measured via CellTrace Violet staining after three days of coculture with treated BM-DCs. Data are the means ± SDs of four independent experiments. Statistical significance was determined using repeated measures one-way analysis of variance (ANOVA) and the *post hoc* Sidak test (*p<0.05; **p<0.01; ns, not statistically significant). **(B, E, F)** Effect of pharmacological inhibitors on FLH and rSIP processing. BM-DCs were pretreated for 2 h with TAK-242 (10 µg/mL) or for 1 h with bafilomycin (10 nM), simvastatin (10 nM), epoxomicin (5 nM), MG132 (4 nM) or DMSO and stimulated for three hours with rSIP + OVA (1 mg/mL) and FLH + OVA (1 mg/mL). Naïve OT-I CD8+ T-cell (2.5 x 10^5^ cells) proliferation was measured via CellTrace Violet (CTV) staining after three days of coculture with treated BM-DCs. For bafilomycin (10 nM), simvastatin (10 nM), epoxomicin (5 nM), and MG132 (4 nM), the data are the means ± SDs of three independent experiments. For TAK-242, the data are the means ± SDs of seven independent experiments. Statistical significance was determined using **(B)** the Mann–Whitney U test and **(E, F)** repeated measures one-way analysis of variance (ANOVA) and the *post hoc* Sidak test (****p<0.0001; ns, not statistically significant). **(C, D)** Dendritic cells were pretreated for 18 h with Pepinh-TRIF or Pepinh-MyD and stimulated for three days with rSIP + OVA (1 mg/mL) and FLH + OVA (1 mg/mL). Naïve OT-I CD8+ T-cell proliferation (2.5 x 10^5^ cells) was measured via CTV staining after three days of coculture with treated BM-DCs. Data are the means ± SDs of seven independent experiments. Statistical significance was determined using the Mann–Whitney U test (*p<0.05; **p<0.01). **(G)** Flow cytometry analyses show representative CTV dilution profiles for the experiments shown in figures **(B–D)**. Representative histograms of CTV dilution in gated CD8+ OT-I cells represent the inhibition of TAK-242, MyD88 and TRIF. ***p<0.001.

After establishing a pattern associated with the recruitment of MyD88 and TRIF by rSIP and FLH for signaling, we decided to characterize the adjuvant effects of these proteins on antigen cross-presentation. Following TLR4-induced maturation of DCs, antigen cross-presentation is first enhanced and then modulated downstream of antigen internalization and cytosolic delivery (36). We wanted to investigate whether these two TLR4 ligands exerted an adjuvant effect on antigen cross-presentation; to this end, we pulsed BM-DCs for 3 h with OVA, OVA + LPS, OVA + FLH, and OVA + rSIP formulations. The BM-DCs were washed with PBS and cocultured for three days with CellTrace Violet (CTV)-labeled naïve CD8+ T cells (OT-I). Dye dilution in proliferative cells was used to characterize the activation of naïve CD8 T lymphocytes based on flow cytometry. FLH and rSIP promoted CD8+ T-cell proliferation compared to control OVA (**Figure 7A**). However, FLH generated an effect at 1 and 0.5 mg/mL, whereas rSIP only did so at 1 mg/mL. Additionally, enhancement of the antigen-specific response induced by both PBAs was revealed by the 25D1.16 mAb antibody that recognizes MHC-I loaded OVA peptide (H-2Kb-SIINFEKL), and at 72 h post-stimulation with rSIP and FLH, there was promoted an increase in the population of CD11c+ 25D1.16+ cells (data not shown). Furthermore, rSIP and FLH induced classical MHC-II presentation to CD4+ T cells from OT-II mice. Their effects were similar to those observed for antigen cross-presentation, with FLH and rSIP enhancing T-cell activation compared to that observed with OVA alone (**Supplemental Figure 2**). Antigen cross-presentation is relevant because it confirms that the protein ligands had characteristics that were different from each other, which could be associated with their molecular structures and their affinities for TLR4.

To determine whether the adjuvant effect of rSIP and FLH was due to TLR4, we pretreated BM-DCs with DMSO or TAK242 to inhibit TLR4 signaling. The results showed that the activation of CD8 T-lymphocytes was inhibited, from approximately 80% to 20%, for both proteins (**Figures 7B, G**). With the recruitment inhibition approach for MyD88 and TRIF, both rSIP and FLH decreased CD8 T lymphocyte proliferation (**Figures 7C, D, G**). Remarkably, FLH stimulated approximately 80% activity in the control, and when MyD88 was inhibited, proliferation fell to approximately 40%. A similar effect was observed with rSIP, with proliferation decreasing from approximately 80% to 45% after inhibition of MyD88 recruitment (**Figures 7C, G**). Similarly, FLH stimulated approximately 40% activity after inhibition of TRIF recruitment, while the control proliferation was up to approximately 80%. An effect similar to that seen for rSIP was observed, with FLH stimulating up to approximately 80% of control cells, and inhibition of TRIF recruitment inducing proliferation in 45% of cells (**Figures 7D, G**).

Since the adjuvant effect of TLR4 on antigen cross-presentation depends on vacuolar processes (38), we decided to use two vacuolar inhibitors, simvastatin and bafilomycin. Pretreatment with these two inhibitors decreased CD8 T lymphocyte proliferation in response to FLH and rSIP (**Figures 7E, G**). In the case of FLH, the DMSO control stimulated proliferation in approximately 80% of CD8 T lymphocytes, while pretreatment with simvastatin and bafilomycin generated values of 10%. In the case of rSIP, DMSO induced proliferation in approximately 80% of cells, while pretreatment with simvastatin and bafilomycin generated values of approximately 16% and 10%, respectively.

Since TRIF is involved in the proteasome pathway associated with antigen cross-presentation, we decided to characterize the effects of proteasomal inhibitors (43). As described for bafilomycin and simvastatin, we pretreated cells with epoximicin (a proteasome inhibitor) and MG132 (a proteasome inhibitor) for one hour and pulsed them with rSIP plus OVA and FLH plus OVA. We characterized antigen cross-presentation through CD8 T lymphocyte proliferation. The proteasomal inhibitor influenced antigen cross-presentation stimulated by rSIP and FLH (**Figure 7F**). In the case of FLH, the DMSO control stimulated proliferation in approximately 95% of CD8 T lymphocytes, while pretreatment with epoxomicin and MG132 generated values of approximately 45%. In the case of rSIP, DMSO induced proliferation in ∼80% of cells, while pretreatment with epoxomicin and MG132 generated values of approximately 38% and 40%, respectively.

Additionally, given the relevance of vacuolar inhibitors in reducing crossover, we decided to characterize the influence of lysosomal proteases and intermediates on endoplasmic reticulum (ER) to Golgi vesicular transport (44). In this context, similar to Bafilomycin and Simvastatin, we pretreated cells with Brefeldin A (an ER-Golgi traffic inhibitor), Leupeptin (a Cathepsin B inhibitor), and Pepstatin A (a Cathepsin D and E inhibitor) for one hour and pulsed them with rSIP plus OVA and FLH plus OVA and characterized antigen cross-presentation through CD8 T lymphocyte proliferation. Cathepsin D and E inhibitors affected SIP- and FLH-stimulated antigen cross-presentation (**Supplemental Figure 3**). In the case of FLH, the DMSO control stimulated proliferation in approximately 90% of CD8 T-lymphocytes, while pretreatment with pepstatin A generated a value of approximately 50%. In the case of rSIP, DMSO induced proliferation in approximately 80% of cells, while pretreatment with Pepstatin generated values of approximately 38%. Conversely, in the case of Cathepsin D, Leupeptin was only significant inhibitor of FLH and generated OT-I lymphocyte proliferation values of 40%. In the case of inhibition of traffic from the endoplasmic reticulum (ER) to the Golgi, Brefeldin A generated CD8 T-lymphocyte proliferation values of 40% and 50% after stimulation with rSIP and FLH, respectively. Together, these results suggest that rSIP and FLH generate an adjuvant effect on antigen cross-presentation and depend on MyD88 and TRIF recruitment. Moreover, vacuolar and cytosolic pathways are essential for these effects on antigen cross-presentation.

## 4 Discussion

Few adjuvants currently used in licensed vaccines are known to elicit potent cytotoxic T-lymphocyte (CTL) responses. Thus, the development of new vaccine adjuvants is considered one of the slowest processes in the history of medicine (1). Nevertheless, the results of several studies are consistent with the idea that modulation of the TLR4 signaling pathway using Lipid A or monophosphoryl lipid A (MPL) can be used to dissociate beneficial immune responses from harmful LPS side effects, which are attributed to the stronger activation of NF-κB than MPL in APCs (45–48), leaving a gap in our understanding of how downstream signaling is affected by different protein agonists of this receptor. Therefore, elucidating the contributions of TLR4 agonist protein adjuvants that modulate proinflammatory activity and immunomodulation will help researchers understand the adjuvant effects of these molecules on the immunological synapse between APCs and T cells.

The rationale for using two model adjuvant proteins, FLH and rSIP, whose use as potential adjuvants has been previously documented through *in vivo* studies in murine models, is based on their similarities and differences. Several similarities have been described: (i) both are TLR4 agonist proteins, (ii) they promote the maturation of DCs, (iii) a limited understanding of the TLR4-associated cell signaling pathway exists, (iv) their contributions to the presentation of exogenous antigens needs to be better understood, and (v) both induce the development of adaptive responses of the Th1 type. Among their differences, two are worth mentioning: (i) species of origin: FLH comes from a mollusk, while rSIP is bacterial, and (ii) the structure of hemocyanin is a very large glycosylated oligomeric protein, unlike rSIP, which is small and lacks oligosaccharides. One of the advantages of TLR4-PBAs is that they can ensure a shared antigen–adjuvant load. rSIP can be expressed in a heterologous system in conjunction with the antigen, while FLH must be conjugated to the antigen. However, it is unknown how rSIP and FLH affect the immune system by binding to TLR4, a receptor that activates multiple signal transduction pathways via MyD88, and TRIF. In this study, we compared these PBAs of TLR4, revealing that their immunomodulatory effects are codependent on MyD88 and TRIF in.

Subunit vaccines containing highly purified recombinant pathogen components are safe; however, they are poorly immunogenic and thus require the use of adjuvants to increase their immunogenicity (42). The protection provided by the most effective vaccines depends on the induction of neutralizing antibodies. Unfortunately, most currently used adjuvants are poorly effective in inducing strong cellular immunity (1, 2, 7). For diseases requiring neutralizing antibodies and T-cell immunity, such as acquired immunodeficiency syndrome (AIDS), severe acute respiratory syndrome coronavirus 2 (SARS-CoV-2), tuberculosis, and malaria, it is essential to incorporate immune adjuvants that elicit strong T-cell immunity (1, 2, 7). To trigger the induction of robust CD8 T-cell immunity by vaccines, it is necessary to engage the antigen processing pathway for cross-presentation by APCs, as previously described (1, 2, 7). Although rSIP and FLH activate TLR4 signaling pathways that depend on MyD88 and TRIF recruitment, both proteins undergo finely tuned regulation of their adjuvant effects, which is associated with the intrinsic molecular properties of each protein. Indeed, although there are no crystallographic data for these proteins, considering the available published data, it is possible to confirm that they are very different, as one is a very large, glycosylated protein with a complex quaternary structure (20), and the other is a small nonglycosylated protein without multiple subunits (49). These differences strongly suggest that the interactions of these proteins with TLR4 could be different. Indeed, the interaction of FLH with TLR4 occurs due to the oligosaccharide residues of FLH (as a viral protein) because when FLH glycosylations are removed, the interaction decreases significantly (23). In contrast, the binding of rSIP could be facilitated by CD14 and the contribution of the MD-2 protein stably associated with the extracellular fragment of the receptor.

The function of DCs stimulated with TLR4 is linked to the greater abundance of costimulatory molecules on their surface, cytokines, and receptors in addition to chemokines and promotes adaptive immunity by activating specific T-lymphocytes. MyD88 signaling is associated with proinflammatory and innate immune responses (50). In contrast, TRIF signaling is associated with the development of an adaptive immune response, which is essential for effective vaccination (10). Although preliminary studies characterizing MyD88 and TRIF interactions with TLR4-LPS have been published (12), in this work, we characterized two protein agonists from different species for the first time. Furthermore, this study establishes that MyD88 and TRIF are essential for the adjuvant effects of these proteins. Specifically, one of the most notable effects is that rSIP and FLH generate IL-6 and IP-10 transcripts in a manner dependent on MyD88 and TRIF. However, in terms of IL-6 and IP-10 secretion, only rSIP depends on MyD88 and TRIF, while FLH is TRIF dependent. These differences can be explained by the fact that the genome-wide correlation between mRNA expression levels has an explanatory power of approximately 40% and can be attributed to other levels of regulation between the transcript and the protein product (51–53).

TLR4 can interact with other pattern recognition receptors (PRRs) to mediate intracellular signaling and interactions with C-type lectin receptors, such as MR and DC-SIGN, to promote, in some cases, antigen cross-presentation (54–56). Following TLR4 agonist-induced DC maturation, processes associated with antigen cross-presentation, such as scavenging receptor-mediated phagocytosis and phagolysosomal fusion, are enhanced during the initial hours of TLR4 activation, after which a loss of antigen internalization and the molecular components necessary for cytosolic delivery of antigen occurs (57). Gupta et al. found that MHC-I molecules are not derived from the endoplasmic reticulum–Golgi intermediate compound (ERGIC) upon TLR stimulation because ERGIC components are recruited to phagosomes independent of TLR signaling (38). However, stimulation of TLR4 results in the accumulation of MHC class I molecules derived from the endocytic recycling compartment (ERC; marked by Rab11a and vesicle-associated membrane proteins 3 and 8 [VAMP3 and 8, respectively]) in phagosomes (44, 58). In addition, TLR-mediated MyD88-dependent IKK phosphorylation of synaptosome-associated protein 23 (SNAP23) mediates endosomal recycling compartment (ERC)–phagosome fusion (38). Alloatti et al. also showed that TLR4 activation delays phagosome maturation and antigen degradation, which induces Rab34-mediated intracellular perinuclear pool formation (36). On the other hand, concerning the endosome-to-cytosol pathway, it is known that the activity of the translocon protein Sec61 in the ER is mediated by TRIF because this step is essential for translocation from the endosome to the cytosol (43). Our results suggest that TLR-based adjuvants likely engage vacuolar pathways to potentiate effective CD8 T-cell responses. However, rSIP and FLH may also be involved in the endosome-to-cytosol pathway via TRIF and the Sec61 protein. This assumption was supported, given that different proteasome inhibitors decreased the proliferation of CD8 OT-I lymphocytes.

This work supports the conclusion that rSIP and FLH mediate TLR4 activation and that this modulation depends on the recruitment of MyD88 and TRIF because each protein can induce finely tuned signaling patterns. This characteristic seemed dependent on the structure of the TLR4 agonist and its potency because FLH can mediate cytokine secretion independent of MyD88 recruitment, and FLH can mediate antigen cross-presentation in a manner dependent on MyD88 recruitment. In contrast, rSIP is totally dependent on MyD88 and TRIF for cytokine secretion and antigen cross-presentation. Notably, the TRIF pathway is essential for rSIP- and FLH-induced secretion of IP-10 and IL-6, and the MyD88 pathway is only essential for rSIP-induced secretion of IP-10 and IL-6 (**Figure 5**). However, IL-6 secretion by FLH was dependent on MyD88 recruitment, which could be attributed to the stimulation time of FLH in BM-DCs, since FLH is influenced by the recruitment of MyD88 during the first 4 hours of stimulation (**Figure 3A**). However, after prolonged stimulation times, the inhibition of MyD88 recruitment did not exert a significant effect on the expression of IL-6 (**Figures 5C**). Another explanation is that the MyD88-adapter-like (MAL) protein could be involved in signaling, as previously described for FLH (17). MAL could be recruited by TRAF6, suggesting that after longer stimulation with FLH, the TRAF6 protein would be activated differently by rSIP, enabling the secretion of IP-10 and IL-6.

**Figure 8:**
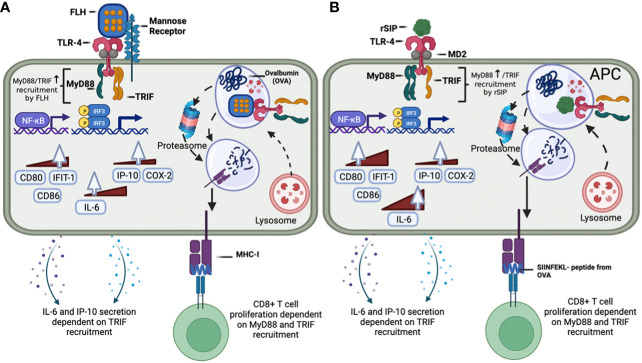
Modulation of the MyD88 and TRIF signaling pathways and downstream responses by the protein-based adjuvants rSIP and FLH in APCs. **(A)** FLH interacts with mannose receptors and activates the TLR4 signaling pathway, which recruits MyD88 and TRIF. FLH activates NF-kB and IRF. MyD88 recruitment is involved in the expression of the IL-6, CD80, IFIT-1, and CD86 mRNAs, while TRIF is involved in the expression of IL-6 and IP-10. Regarding the secretion of cytokines, only TRIF is involved in the secretion of IL-6 and IP-10. FLH then promotes OVA cross-presentation, and its effect is dependent on MyD88 and TRIF. **(B)** rSIP activates the TLR4 signaling pathway, which recruits MyD88 and TRIF. rSIP activates NF-kB and IRF. MyD88 recruitment is involved in expression of the IL-6, COX-2, CD80, IFIT-1, and CD86 mRNAs, while TRIF is involved in the expression of IL-6, COX-2, and IP-10. Regarding the secretion of cytokines, both MyD88 and TRIF are involved in the secretion of IL-6 and IP-10. rSIP then promotes OVA cross-presentation, and its effect is dependent on MyD88 and TRIF.

Regarding the regulation of TLR4, it was previously shown using iterative mathematical models that the pathways mediated by MyD88 and TRIF provide are dependent on the concentrations of ligands that transmit information about the threat of the pathogen (59). These changes in signaling are supported by the fact that the start of TLR4 signaling involves oligomerization, which determines MyD88 and TRIF signaling (60). This implies that one pattern recognition receptor is activated by different microenvironmental cues to generate macrophages with distinct phenotypes linked to a subset of cytokines and phosphoproteomic signaling patterns (61). In this context, our results are consistent with this finding because a lower concentration of rSIP than FLH is needed to activate TLR4. This difference is directly related to the molecular characteristics of each protein (**Figure 8**). Furthermore, this signaling change is supported by the start of TLR4 signaling during dimerization and the oligomerization dynamics, which determines MyD88 and TRIF signaling. Therefore, since rSIP and FLH are partial agonists, their interaction with TLR4 could also be involved in the oligomerization dynamics of this receptor.

In conclusion, these results provide further insight into the nature of TLR4 agonist protein adjuvants and their contributions to activation of the MyD88 and TRIF signaling pathways. These results are relevant since they contribute to our knowledge of how protein-based agonists of TLR4 can act as adjuvants, information that supports the use of these agonists in the development of future experimental vaccines for cancer, persistent diseases, or future pandemics; an ongoing challenge related to controlling the doses of vaccine adjuvants, such as the sMLA adjuvant, which is the active component of the glucopyranosyl lipid adjuvant (GLA), exists (62, 63). Additional studies are needed to establish a preclinical model and determine the effects of these adjuvants and their contributions to the MyD88 and TRIF signaling pathways downstream of TLR4.

## Data availability statement

The original contributions presented in the study are included in the article/**Supplementary Material**. Further inquiries can be directed to the corresponding authors.

## Ethics statement

The animal study was reviewed and approved by Comite Institucional de Cuidado y Uso de Animales de Laboratorio (CICUAL) del Instituto de Salud Pública de Chile (ISPCh).

## Author contributions

DD-D, MIB, and AEV: conception and design. DD-D, MS, DE, BC, BV, and MT: experiment execution and data acquisition. DD-D, MS, AM, FS, SL, JD, MIB, and AEV: data analysis and interpretation. DD-D, MIB, and AEV: manuscript writing. All authors contributed to the article and approved the submitted version.

## Supplementary material

Supplementary Figure 1 Partial and differential agonism of Toll-like receptors 4 (hTLR4) by protein-based adjuvants. **(A)** Determination of the concentrations of protein agonists needed to activate hTLR4. HEK-Blue-hTLR4 reporter cells were exposed to different concentrations of protein adjuvants. Dose−response curves were generated for cells exposed to a maximum concentration of 2,540 nM rSIP and FLH for 48 h. Data show normalized HEK-Blue mTLR4 cell responses considering LPS treatment as 100% stimulation; 100% = maximum dose plateau of the LPS agonist. **(B)** Comparison of the log EC_50_ values of protein agonists in activating mTLR4. Log (EC_50_) values for rSIP and FLH were determined according to the relative abundance of soluble AP secreted by Hek-Blue-hTLR4 cells. Individual log (EC_50_) values and mean values from three independent experiments are shown. The statistical significance of differences was analyzed using an unpaired t test (ns: not significant). **(C)** IRF3 activation by FLH and rSIP in BM-DCs. Phosphorylation of IRF3 induced by FLH and rSIP in BM-DCs determined by Western blotting. BM-DCs from 6 mice (pool) were used for analysis of phospho-IRF-3 (Ser396) (4D4G). As a positive control, A549 cells stimulated with poly (I:C) (C+) and without stimulation (C-) were used. The BM-DCs were stimulated for 0, 1, 2, 7, 10, 13, and 18 hours.

Supplementary Figure 2 rSIP and FLH induce classical antigen presentation. BM-DCs were stimulated for 18 h with rSIP and FLH and coadministered OVA (1 mg/mL). **(A)** The percentage and **(B)** MFI of naïve OT-II CD4^+^ T-cell (3x10^5^ cells) activation (CD69+) were measured after 18 h of coculture with treated BM-DCs. Data are the means ± SDs of three independent experiments. Statistical significance was determined by one-way analysis of variance (ANOVA) with Tukey’s multiple comparisons test (*p<0.05; ns: statistically not significant). **(C)** Flow cytometry analyses show representative CD4+ CD69+ profiles of OT-II cells cocultured with stimulated BM-DCs.

Supplementary Figure 3 rSIP and FLH induce antigen cross-presentation through cathepsin. BM-DCs were pretreated for 1 h with DMSO, brefeldin A (1 µM), leupeptin (10 µM), and pepstatin A (40 nM) and stimulated for three hours with rSIP + OVA (1 mg/mL) and FLH + OVA (1 mg/mL). Naïve OT-I CD8+ T-cell (2.5x10^5^ cells) proliferation was measured via CellTrace Violet staining after 3 days of coculture with treated BM-DCs. Data are the means ± SDs of three independent experiments. Statistical significance was determined using repeated measures one-way analysis of variance (ANOVA) and the *post hoc* Sidak test (*p<0.05; **p<0.01; ns: statistically not significant).
