# Supplementary figures and images for: Surface immunogenic protein from Streptococcus agalactiae and Fissurella latimarginata hemocyanin are TLR4 ligands and activate MyD88- and TRIF dependent signaling pathways
Source: Front Immunol. 2023 Sep 18;14:1186188. doi: 10.3389/fimmu.2023.1186188 (PMC10544979; doi:10.3389/fimmu.2023.1186188)

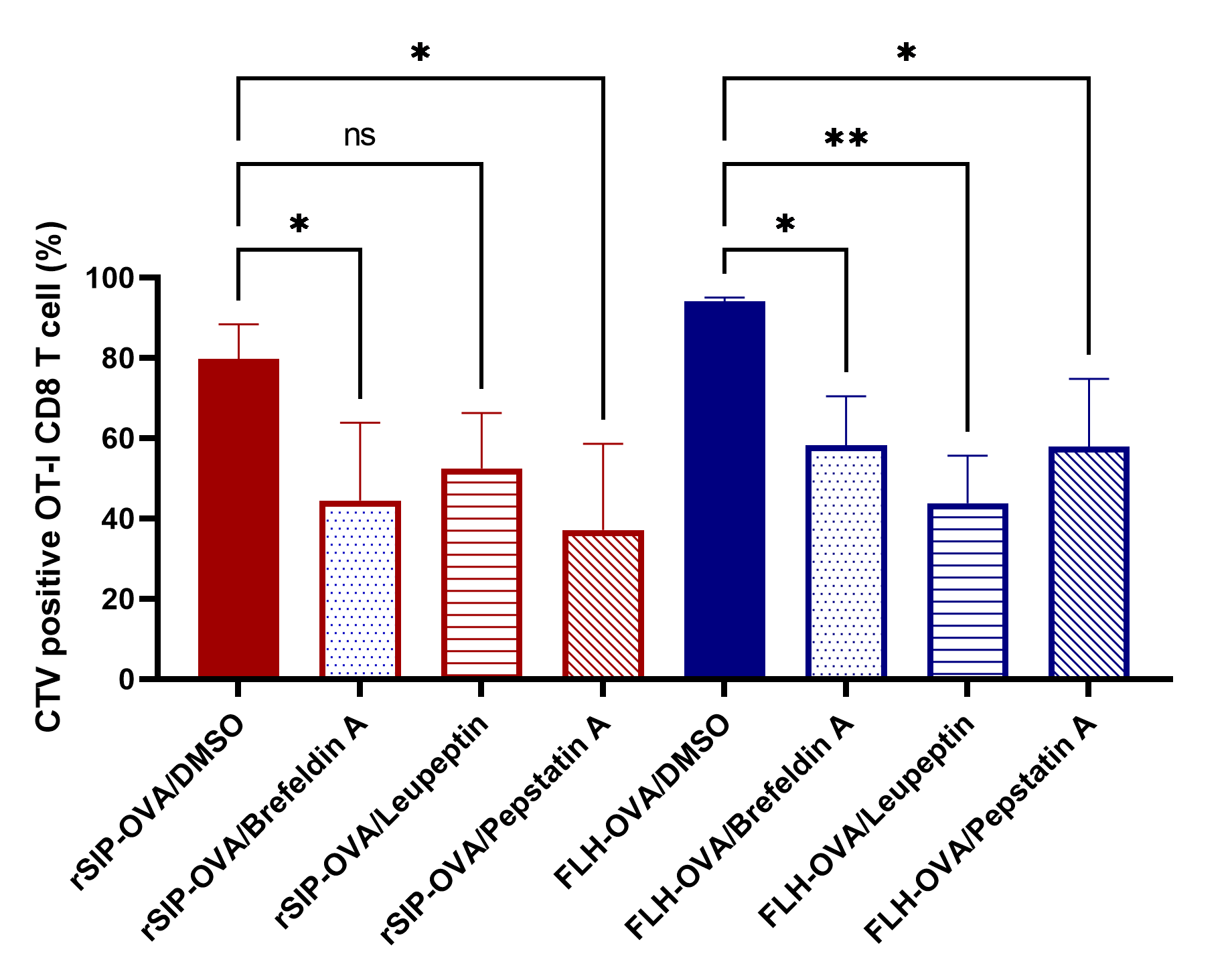

Supplement: Supplementary Figure 3 — rSIP and FLH induce antigen cross-presentation through cathepsin. BM-DCs were pretreated for 1 h with DMSO, brefeldin A (1 µM), leupeptin (10 µM), and pepstatin A (40 nM) and stimulated for three hours with rSIP + OVA (1 mg/mL) and FLH + OVA (1 mg/mL). Naïve OT-I CD8+ T-cell (2.5x10^5^ cells) proliferation was measured via CellTrace Violet staining after 3 days of coculture with treated BM-DCs. Data are the means ± SDs of three independent experiments. Statistical significance was determined using repeated measures one-way analysis of variance (ANOVA) and the post hoc Sidak test (*p<0.05; **p<0.01; ns: statistically not significant). [file Image_3.tif]
