# Supplementary material for: Surface immunogenic protein from Streptococcus agalactiae and Fissurella latimarginata hemocyanin are TLR4 ligands and activate MyD88- and TRIF dependent signaling pathways
Source: Front Immunol. 2023 Sep 18;14:1186188. doi: 10.3389/fimmu.2023.1186188 (PMC10544979; doi:10.3389/fimmu.2023.1186188)
Supplement: Supplementary file 4 [file Table_1.docx]

**Supplementary table 1.**

| Number | Name | Type | Secuence |
| --- | --- | --- | --- |
| 1 | Mus_musculus_interleukin 6_FW | Forward Primer | ATAAGCTGGAGTCACAGAAGG |
| 2 | Mus_musculus_interleukin 6_RV | Reverse Primer | CACTAGGTTTGCCGAGTAGAT |
| 3 | Mus_musculus_interleukin 6_PROBE | Probe | FAM-TTGGATGGTCTTGGTCCTTAGCCAC-BHQ1 |
| 4 | Prostaglandin-endoperoxidase_2_FW | Forward Primer | CGGACTGGATTCTATGGTGAAA |
| 5 | Prostaglandin-endoperoxidase_2_FW | Reverse Primer | CTTGAAGTGGGTCAGGATGTAG |
| 6 | Prostaglandin-endoperoxidase_2_PROBE | Probe | FAM-TCAAATTACTGCTGAAGCCCACCC-BHQ1 |
| 7 | CD80_FW | Forward Primer | GGCTCTAGATTCCTGGCTTTC |
| 8 | CD80_RV | Reverse Primer | GGAAACTTGAGGAGTGGTGTAT |
| 9 | CD80_PROBE | Probe | FAM-TGGCTTGCAATTGTCAGTTGATGCA-BHQ1 |
| 10 | CD86_FW | Forward Primer | TGGGCACAGAGAAACTTGATAG |
| 11 | CD86_RW | Reverse Primer | TCTGAACATTGTGAAGTCGTAGAG |
| 12 | CD86_PROBE | Probe | FAM-ACGAGCTTTGACAGGAACAACTGGA-BHQ1 |
| 13 | IP10_FW | Forward Primer | AGTAACTGCCGAAGCAAGAA |
| 14 | IP10_RV | Reverse Primer | GCACCTCCACATAGCTTACA |
| 15 | IP10_PROBE | Probe | FAM-TGGGATGGCTGTCCTAGCTCTGTA-BHQ1 |
| 16 | IFIT1_FW | Forward Primer | CACCAGTATGAAGAAGCAGAGAG |
| 17 | IFIT1_RV | Reverse Primer | GCCATAGCGGAGGTGAATATC |
| 18 | IFIT1_PROBE | Probe | FAM-AGGCACTGAACAACAAGACCCTCG-BHQ1 |
| 19 | Actb-fw | Forward Primer | TTTCCAGCCTTCCTTCTTGG |
| 20 | Actb-rv | Reverse Primer | GGCATAGAGGTCTTTACGGATG |
| 21 | Actb-probe | Probe | **HEX-**TGGAATCCTGTGGCATCCATGAAACT**-BHQ2** |
